# Design and Development of a Smart IoT-Based Robotic Solution for Wrist Rehabilitation

**DOI:** 10.3390/mi13060973

**Published:** 2022-06-19

**Authors:** Yassine Bouteraa, Ismail Ben Abdallah, Khaled Alnowaiser, Md Rasedul Islam, Atef Ibrahim, Fayez Gebali

**Affiliations:** 1 Department of Computer Engineering, College of Computer Engineering and Sciences, Prince Sattam Bin Abdulaziz University, Al-Kharj 11942, Saudi Arabia; k.alnowaiser@psau.edu.sa (K.A.); aa.mohamed@psau.edu.sa (A.I.); 2 Control and Energy Management Laboratory (CEM Lab.), Ecole Nationale d Ingenieurs de Sfax (ENIS), Institut Superieur de Biotechnologie de Sfax (ISBS), University of Sfax, Sfax 3038, Tunisia; ismail.benabdallah@enis.tn; 3 Richard J. Resch School of Engineering, University of Wisconsin-Green Bay, Green Bay, WI 54311, USA; islamm@uwgb.edu; 4 Electrical and Computer Engineering Department, University of Victroia, Victoria, BC V8P 5C2, Canada; fayez@uvic.ca

**Keywords:** wrist modeling, robotic rehabilitation, human robot interaction

## Abstract

In this study, we present an IoT-based robot for wrist rehabilitation with a new protocol for determining the state of injured muscles as well as providing dynamic model parameters. In this model, the torque produced by the robot and the torque provided by the patient are determined and updated taking into consideration the constraints of fatigue. Indeed, in the proposed control architecture based on the EMG signal extraction, a fuzzy classifier was designed and implemented to estimate muscle fatigue. Based on this estimation, the patient’s torque is updated during the rehabilitation session. The first step of this protocol consists of calculating the subject-related parameters. This concerns axis offset, inertial parameters, passive stiffness, and passive damping. The second step is to determine the remaining component of the wrist model, including the interaction torque. The subject must perform the desired movements providing the torque necessary to move the robot in the desired direction. In this case, the robot applies a resistive torque to calculate the torque produced by the patient. After that, the protocol considers the patient and the robot as active and all exercises are performed accordingly. The developed robotics-based solution, including the proposed protocol, was tested on three subjects and showed promising results.

## 1. Introduction

Stroke is considered to be one of the leading causes of death worldwide, according to the World Health Organization. Indeed, each year, more than 15 million people in the world are victims of a stroke [1]. Those who survive and move past this crisis may have general limitations, such as problems with movement and speech, as well as changes in their thinking and feelings. In order to reduce the damage and cope with the catastrophic consequences of a stroke, rehabilitation must take place as soon as possible. Indeed, post-stroke rehabilitation can stimulate plasticity as well as prevent and treat problems. The initial goal is to improve brain flexibility through brain stimulation, which reorganizes healthy neural networks to ensure that missing neurons play the maximum possible role in brain necrosis. This rehabilitation work has been carried out for decades by physiotherapists manually or semi-automatically and the rehabilitation has been largely based on repetitive movements aimed at recovering lost motor function. Hence, the idea of automating these exercises and taking advantage of huge technological advances to gain efficiency and increase productivity. In this context, robots are essential in the rehabilitation process to promote the motor recovery of post-stroke participants. Indeed, robots can step up treatment, aid patient movement, and provide feedback. Systems dedicated to automated rehabilitation have benefited from numerous technological advances in robotics and information technologies [2,3,4,5,6]. In this context, we aim to take advantage of the progress made in mathematical modeling of human joints, the IoT-based system design, fuzzy logic-based decision-support systems to design a new wrist rehabilitation protocol based on a human wrist model. Understanding the dynamics of the wrist joint is very important in the field of rehabilitation. Indeed, the mathematical model of the wrist joint dynamics is very useful for characterizing the torque necessary to overcome the passive mechanical impedance of wrist rotation. The motion equations of wrist motions rely on many parts including inertia, damping, and stiffness [7,8]. These parameters are related to the subject and can be determined by experimental measurements. In this work, a mathematical model of the dynamics of wrist rotations in two degrees of freedom (DOF) is developed and integrated into the control scheme, evaluating the torques required for wrist rotations and establishing the relative importance of the different terms and parameters of the model. These torques produced by the wrist joint are very sensitive to muscle fatigue. In order to overcome this constraint, an EMG-based muscle fatigue estimation system is developed. Actually, EMG has been widely used in robotic applications [9,10,11,12,13,14]. Based on EMG signals, a myoelectrical driven robotic system has been proposed for elbow training assistance [10]. In [12], the authors created a virtual reality-based rehabilitation training system using EMG feedback. A novel notion of robotic therapy based on EMG thresholds was presented in [13]. In [14], an overview of EMG-based control of human upper limb movements was presented. In addition, based on the obtained EMG rows, a fuzzy classifier has been used to evaluate muscle exhaustion. Actually, due to its ability to model uncertainty and ambiguous input, fuzzy control has been widely used in many robotic applications [15,16]. In most fuzzy robotic applications, sensors are used to obtain information from uncertain environments to be used as inputs for the fuzzy controller which controls the behavior of the robot through the use of linguistic descriptions modeled using a rules database. In this research, the proposed rehabilitation protocol uses a fuzzy logic controller to describe muscle exhaustion and update the wrist model throughout rehabilitation therapy. The fuzzy controller allows the system to react quickly and reliably to changes in rehabilitation circumstances, including the patient’s condition. Using the Internet of Things (IoT) for control is becoming increasingly popular. Through the IoT concept, system parts and objects are accessible from anywhere and at any time. Incorporating connected robots into the rehabilitation process sounds impressive as it improves overall efficiency, reduces expenses, and motivates patients to participate in the process. By limiting patient visits to clinics, remote robotic rehabilitation can help to reduce stress in healthcare facilities. Therapists will be able to control and monitor their patients at home using tele-rehabilitation technologies. As far as the market is concerned, rehabilitation systems are extremely large, bulky, immobile, and expensive. Due to the fact that the idea is intended to design small robots with a well-defined target that can be used at home, the proposed robotic solution is lightweight and portable as compared with earlier works [2,3,4,5]. Additionally, we propose a new design that reduces the number of motors in the robot. In fact, the particularity of this design lies in its ability to support two movements around two distinct axes using a single actuator. There have been several publications on robotic rehabilitation that do not consider a human joint model in their robot control architecture [17,18,19]. Other studies use muscular contraction detection to quantify fatigue [20,21]. A sensor fusion approach is presented to evaluate the degree of fatigue in this established control architecture. During therapy, a cascaded fuzzy logic controller uses the output of the fuzzy classifier to calculate the patient’s joint model and update it based on the weariness level of the patient. In this study, a new rehabilitation protocol is presented. The first step of this protocol consists of calculating the subject-related constant parameters. This concerns axis offset, inertial parameters, passive stiffness, and passive damping. Axis offset is defined as a constant and the inertial parameters are measured directly from subjects. Passive stiffness and damping were measured from the first exercise where the force and position were recorded from the totally passive patient. The second step consists of determining the remaining components of the wrist model including the interaction torque. The subjects must perform the desired movements providing the torque necessary to move the robot in the desired direction. In this case, the robot applies a resistive torque to calculate the torque produced by a patient. However, this step concerns only the subjects which are in the middle or high level of rehabilitation progress. Indeed, subjects who cannot provide a minimum readable torque begin with passive exercises and their wrist model contains only passive components. In addition, generated torque is a time variant parameter due to muscle fatigue. Therefore, fatigue estimation must be performed based on a reliable natural interface such as EMG signals. In future exercises, total torque is the sum of the torque produced by the robot and the torque, if any, provided by patient taking into consideration the fatigue constraints. There are two main goals of wrist modeling, the first is to evaluate the rehabilitation state level and, the second is to extract the main components of a wrist dynamic model.

## 2. Methodology

### 2.1. Mechanical Design

**Figure 1:**
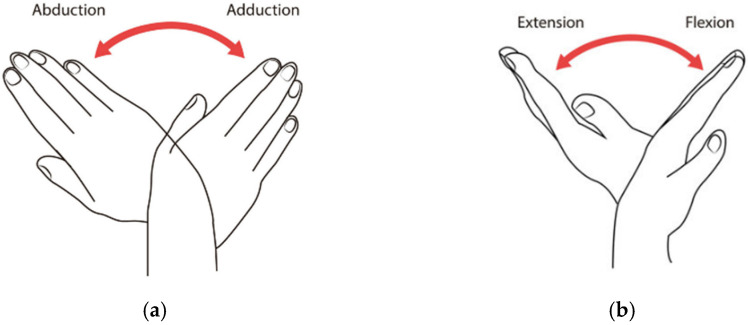
(**a**) Abduction/adduction wrist movements; (**b**) flexion/extension wrist movements.

**Figure 2:**
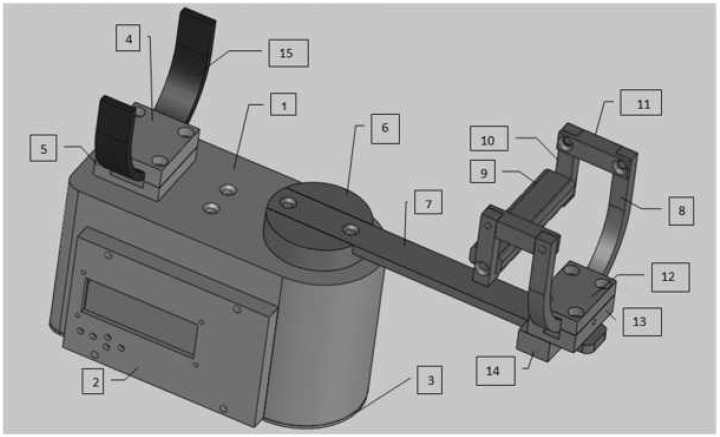
3D design of the robot.

The mechanical structure was designed by considering the anatomical constraints of the wrist movements presented in Figure 1. Figure 2 illustrates the CAD design of the robot including the main components of the assembly listed in Table 1. Physical constraints were also considered in the design ensuring a reduced size and a light weight. 

The model contains five main blocks. Each block is described with its elementary components in Table 1. The sliding bar (Piece 7) is attached to the drive disk (Piece 6) assembling block “B”, while the drive disk is fixed to the motor shaft. 

**Figure 3:**
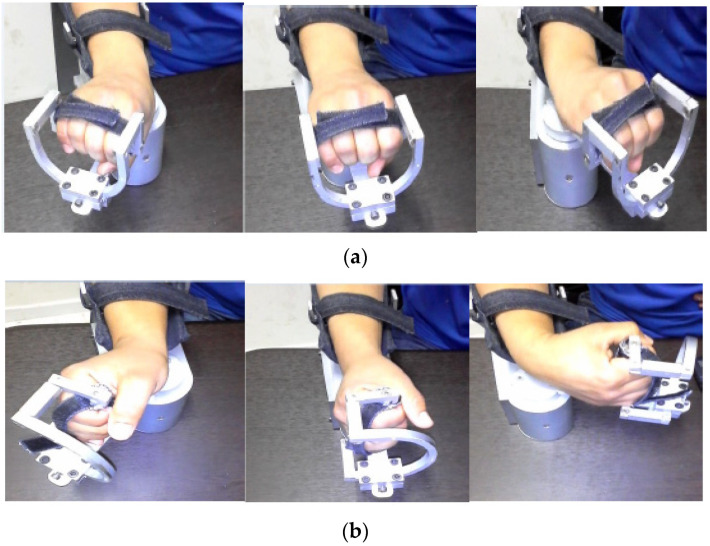
(**a**) Abduction/adduction movement; (**b**) Flexion/extension movement.

Unlike similar solutions, the robot is powered by a single motor performing the two main degrees of freedom (DOF) of the wrist joint. We offer a new solution providing 2 DOF using a single motor to power the joint instead of double motors. Based on a flexible mechanical transformation, the robot ensures the two fundamental movements of the wrist: abduction/adduction and flexion/extension. In fact, the hand support part can be reconfigured moving from the horizontal position (producing the abduction/adduction derivation) to the vertical position (producing the flexion/extension derivation). Indeed, the current state, presented in Figure 3a, ensures the abduction-adduction movement. Removing the higher front arc support in block “D”, the subject can rotate block “C” to switch the other wrist movement providing the flexion/extension movement (Figure 3b).

Figure 3 presents the mechanical transformation ensuring the two fundamental wrist movements. The proposed wrist robot is practical and easy to use. In fact, it is a portable device that is not complicated at all and did not need specific training to configure it. In addition, the proposed robot is actuated by a high-torque servo-stepper motor providing the required torque to apply the new rehabilitation protocol.

### 2.2. Second-Order Mechanical Impedance Model of Wrist Rotations

In this section, we present a mathematical model of wrist rotation dynamics in the two fundamental DOF, evaluating the torques required for wrist rotations, and determining the relative importance of various model terms and parameters in order to develop a complete model useful for rehabilitation exercises. The model is evaluated using experimentally observed passive wrist movements imposed by a rehabilitation robot to define the dynamics of wrist rotations. It is worth noting that stiffness, not inertia, dominates wrist rotation dynamics and that, unlike the arm, the wrist DOF are linked by stiffness rather than inertia. We show that the equations of motion are accurately approximated as linear for moderately sized wrist rotations, implying that a very basic internal model could offer a sufficiently accurate estimate of the torques required to govern wrist rotations in rehabilitation exercises.

**Figure 4:**
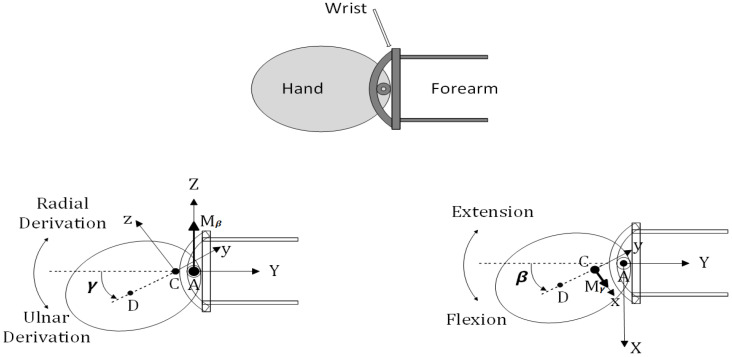
Diagram of the actual wrist and universal joint model.

The wrist joint can be described kinematically as a universal joint with non-intersecting axes (Figure 4). Flexion–extension occurs about the Z axis and is represented by *β*, and radial–ulnar deviation occurs about the rotated x axis and is represented by *γ*. A number of torques are applied to the hand when the wrist rotates. Muscle contraction produces active torques. Here, we present them as a group and resolve them (with no loss in generality) along the flexion/extension (FE) and radial/ulnar derivation (RUD) axes (Figure 4). Passive torques are due to passive mechanical properties of the hand and wrist (inertia, damping, stiffness, etc.) and to gravity. The two-dimensional torque fields resulting from FE and RUD are a combination of passive and active torques. RUD may be torqued by a displacement in FE, and vice versa. To simplify the calculation, damping and stiffness torque fields are assumed to be linear and symmetric about the neutral wrist position, which results in damping and stiffness tensors that are 2-by-2 symmetric. The wrist joint model is illustrated in Figure 4. In the model, the space-fixed XYZ reference frame is fixed in the forearm.

The body-fixed XYZ frame is fixed in the hand (third metacarpal) and rotates with the hand. Flexion–extension occurs about the Z axis and is represented by *β*, and radial–ulnar deviation occurs about the rotated x axis and is represented by *γ*.

The equations of motion of a universal joint with non-intersecting axes, subject to the abovementioned torques, are as follows (Figure 4):

$$T_{\beta }=T_{inert,\beta }+T_{damp,\beta }+T_{stiff,\beta }\;$$

(1) $$T_{\gamma }=T_{inert,\gamma }+T_{damp,\gamma }+T_{stiff,\gamma }+T_{grav,\gamma }\;$$

where

$$\begin{array}{c} T_{inert\;,\beta }=\ddot{\beta }[I_{w}+I_{y}\sin^{2}\gamma +I_{z}\cos^{2}\gamma +mr_{AC}\left(r_{AC}+2r_{CD}\cos\gamma \right)]\\ +\dot{\beta }\dot{\gamma }\left[2\left(I_{y}-I_{z}\right)\sin\gamma \cos\gamma -2mr_{AC}r_{CD}\sin\gamma \right] \end{array}\;\;T_{inert,\gamma }=\ddot{\gamma }I_{x}-{\dot{\beta }}^{2}\left[\left(I_{y}-I_{z}\right)sin\gamma cos\gamma -mr_{AC}r_{CD}sin\gamma \right]\;\;T_{damp,\beta }=B_{\beta \beta }\dot{\beta }+B_{\beta \gamma }\dot{\gamma }\;\;T_{damp,\gamma }=B_{\gamma \gamma }\dot{\gamma }+B_{\beta \gamma }\dot{\beta }\;\;T_{stiff,\beta }=K_{\beta \beta }\beta +K_{\beta \gamma }\gamma \;\;T_{stiff,\gamma }=K_{\gamma \gamma }\gamma +K_{\beta \gamma }\beta \;\;T_{grav,\gamma }=-mgr_{CD}cos\gamma \;$$

FE and RUD are represented by β and γ; $T_{\beta }$ and $T_{\gamma }$ are dynamic torques developed by muscle contraction; the inertia moment of the wrist link around the Z axis at point A is represented by $I_{w}$; while the hand inertia moments around the x, y, and z axes at point C are represented by $I_{x}$, $I_{y}$, and $I_{z}$, respectively; hand mass is denoted by m; axis offset is denoted by $r_{AC}$; the distance between the hand center of mass and the distal axis is denoted by $r_{CD}$; $B_{\beta \beta }$, $B_{\beta \gamma }$, and $B_{\gamma \gamma }$ are elements of the damping tensor; $K_{\beta \beta }$, $K_{\beta \gamma }$, and $K_{\gamma \gamma }$ are elements of the stiffness tensor; and g is the gravitational constant. 

In each equation, the first term is called the main torque and the second term is called the interaction torque since it depends on the movement of the other degrees of freedom (for example, the second term in $T_{inert,\beta }$ involves $\dot{\gamma }$). The inertial interaction moment is only partially due to the fact that the axes do not intersect. The other part (which does not disappear with $r_{AC}$) is a phenomenon caused by rotation around a non-parallel axis. The stiffness–damping interaction moment is completely independent of the axis offset, but is caused by the stiffness–damping force field not being aligned with the axis of rotation. Despite wrist and hand impedance, Equation (1) maps wrist motion to torques required to cause motion. To follow a trajectory specified by β and γ (and their derivatives), the wrist’s muscles must generate torques $T_{\beta }$ and $T_{\gamma }$ to overcome the combined impedance of the individual inertial, damping, stiffness, and gravity terms in Equation (1). Model parameter values must be accurate in order to quantify these torques.

The parameters of the model are as follows:

**Axis offset** Based on the related literature [22], $r_{AC}$ determines the perpendicular distance between FE and RUD axes. Specifically, the flexion axis measured 3.9 ± 2.0 mm, and the extension axis measured 3.9 ± 1.4 mm. All subjects were given $\;r_{AC}=4\;\mathrm{mm}$.

**Inertial parameters** As stated in the related literature [23], subject, mass, inertia, and center of gravity were each calculated by using segment lengths and inertial parameter equations. The wrist link has a very little inertia $I_{W}$ as compared with $I_{z}cos^{2}\gamma$, and therefore, was overlooked.

**Passive stiffness** The passive stiffness was measured directly from the wrist joint of each subject using the wrist rehabilitation robot in a similar way to the protocol defined by [24]. In this exercise, the relevant patient was asked to relax while the robot slowly rotated the wrist into a custom joint position from neutral in FE and RUD. Force and displacement were recorded. The stiffness in each direction was computed as the slope of a linear approximation of the force–displacement relationship in that direction. 

**Passive damping** The passive viscosity of the wrist joint [25] was quantified only in FE. To calculate the damping tensor [26], we assumed that it was proportional to the stiffness tensor for each subject. The proportionality constant was chosen to give a damping value of 0.03 Nms/rad in pure FE [25].

### 2.3. Control Architecture Design

**Figure 5:**
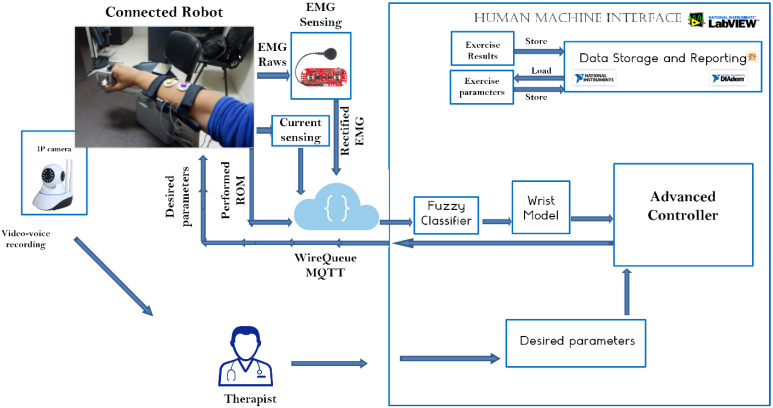
Control architecture overview.

As shown in Figure 5, the main architecture contains several subsystems. The flow starts from the physiotherapist who inputs the desired parameters into the LabVIEW-based human–machine interface (HMI). This HMI contains an advanced controller based on the implemented wrist model. Model parameters are measured and real-time updated during the experiment based on the fuzzy classifier. 

**Figure 6:**
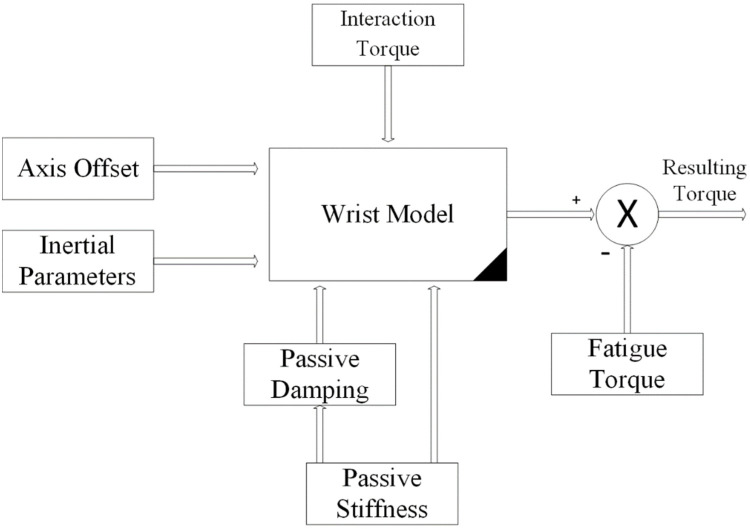
Wrist model and resulting torque.

**Figure 7:**
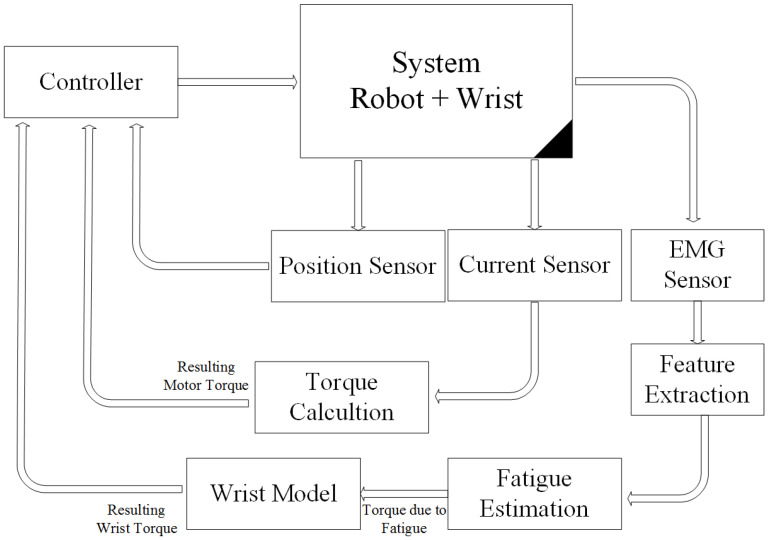
Loaded robot control architecture.

**Figure 8:**
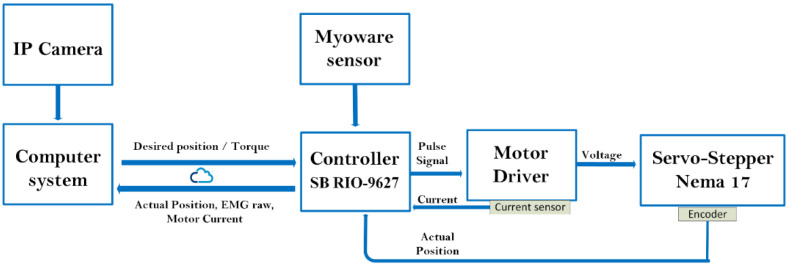
Wrist joint control.

As shown in Figure 6, the wrist model is updated in real time in order to compute the resulting torque produced by the wrist. The resulting torque can be calculated by subtracting the model-based output torque by the estimated torque due to fatigue, as shown in Figure 7. The fatigue estimation block takes the extracted features of the acquired EMG rows as inputs, and then outputs the muscle fatigue based on the fuzzy classifier. On the other hand, motor torque is calculated based on the implemented current sensor and the current joint position of the wrist which is acquired by the related encoder of the servo-stepper motor. As illustrated by Figure 7, the main controller processes the inputs, including the current wrist joint position, the resulting wrist torque and the produced motor torque, and sends the new desired parameters to the robot. In order to apply the new desired parameters, an sbRIO-9627 real-time is responsible for controlling the robot, ensuring motor control, real-time acquisition of the motor current from the current sensor to compute the produced torque, and real-time acquisition and processing of the EMG signals. The sbRIO9627 is an embedded controller that combines a real-time CPU running NI Linux Real-Time, a user-configurable FPGA, and I/O on a single, printed circuit board (PCB). Figure 8 presents the control architecture (acquisition and actuating) of the rehabilitation robot.

#### 2.3.1. EMG Feature Extraction

Feature extraction is the process of extracting relevant information from EMG data and discarding the rest. Signal processing and pattern matching methods, such as the extraction of features and pattern classification, are created in order to appropriately extract the information provided by the EMG signal. The properties utilized to represent the obtained signals have a direct impact on these algorithms. Time domain (TD), frequency domain (FD), and time-frequency or timescale (TS) representation are the primary aspects in an EMG signal analysis [27]. Signal processing and pattern matching methods, such as the extraction of features and pattern classification, are created in order to appropriately extract the information provided by the EMG signal.

Frequency or spectral domain characteristics are commonly utilized to investigate muscle fatigue and MU recruitment. In the frequency domain, power spectral density (PSD) becomes an important analysis. In this part, four frequency-domain feature extraction methods are used as inputs for the fuzzy classifier including the mean frequency, mean power, frequency ratio, and power spectrum ratio. 

Mean frequency (*MNF*) is an average frequency computed by dividing the whole sum of the spectrum intensity by the product of the EMG power spectrum and the frequency [28]. It can be calculated using the formula:

(2) $$MNF=\overset{M}{\underset{j=1}{\sum }}f_{j}P_{j}\;/\;\overset{M}{\underset{j=1}{\sum }}P_{j}$$

where $f_{j}$ is frequency of the spectrum at frequency bin *j*, $P_{j}$ is the EMG power spectrum at frequency bin *j*, and *M* is length of the frequency bin.

Mean power (*MNP*) is an average power of the EMG power spectrum. The calculation is defined as:

(3) $$MNP=\overset{M}{\underset{j=1}{\sum }}P_{j}\;/\;M\;$$

The frequency ratio (*FR*) is proposed to distinguish between contraction and relaxation of muscle using the ratio between the low frequency components and the high frequency components of the EMG signal [29]. The equation is defined as:

(4) $$FR=\overset{ULC}{\underset{j=LLC}{\sum }}P_{j}\;/\;\overset{UHC}{\underset{j=LHC}{\sum }}P_{j}$$

where *ULC* and *LLC* are the low-frequency band’s upper and lower cutoff frequencies, respectively; and *UHC* and *LHC* are the high-frequency band’s upper and lower cutoff frequencies, respectively. There are two techniques to determine the threshold for separating low and high frequencies. The frequency bands are first determined by experiments. The high and low frequency bands can also be set using the MNF feature value [30]. The inverse instance of the high-to-low ratio (H/L ratio) characteristic, which is commonly employed in diaphragmatic fatigue research, is the FR feature.

The power spectrum ratio (*PSR*) is a variation of the PKF and FR characteristics [7]. The PSR is defined as the ratio between the energy *P*_0_, which is close to the EMG power spectrum’s greatest value, and the energy *P*, which is the EMG power spectrum’s total energy. Its formula can be expressed as:

(5) $$PSR=\frac{P_{0}}{P}=\overset{f_{0}+n}{\underset{j=f_{0-n}}{\sum }}P_{j}\;/\;\overset{M}{\underset{j=1}{\sum }}P_{j}$$

where $f_{0}$ is a feature value of the PKF and *n* is the integral limit. 

#### 2.3.2. Fuzzy Classifier Algorithm for Fatigue Estimation

**Figure 9:**
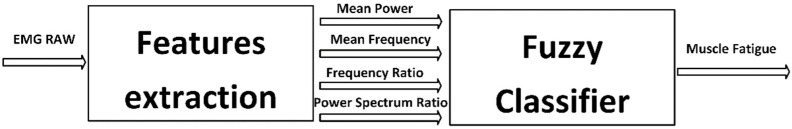
Muscle fatigue estimation.

**Figure 10:**
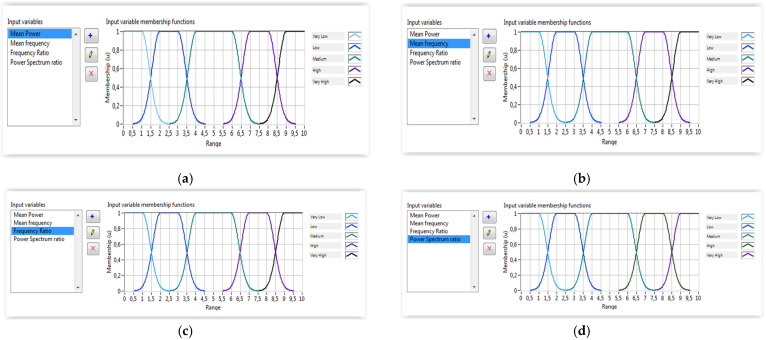
Inputs fuzzy membership functions: (**a**) EMG-MP; (**b**) EMG-MF; (**c**) EMG-FR; (**d**) EMG-FSR.

**Figure 11:**
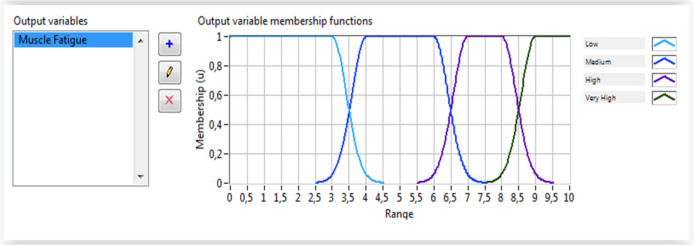
Muscle fatigue estimation.

As discussed above, the wrist torque is mainly based on the wrist model. However, the produced torque is very sensitive to muscle fatigue. Therefore, the torque due to the muscle fatigue is subtracted from the model-based computed torque to determine the real resulting torque. To guarantee a reliable wrist torque calculation, the fatigue torque must be computed experimentally directly from natural muscle interfaces. To achieve this goal, a fuzzy classifier was designed and implemented using LabVIEW software. Indeed, four inputs, generated from the feature extraction algorithm, are used as inputs of the fuzzy classifier which outputs the muscle fatigue level. Figure 9 illustrates the muscle fatigue assessment system. The proposed fuzzy system uses the extracted features of the EMG signals including the mean power, the mean frequency, the frequency ratio and the power spectrum ratio to estimate the degree of muscle fatigue during rehabilitation exercises. The membership functions for the inputs and output are shown in Figure 10 and Figure 11, respectively. The Gaussian membership functions were found to be the most suitable types to be used in the implemented system. The Center of Area (CoA) approach was used as a defuzzification method. The rules database was implemented using LabVIEW software. 

#### 2.3.3. Protocol Description

There are three main phases of the proposed rehabilitation protocol according to the current state of both the robot and the patient where:

**Phase one** The first cycle, robot is active and patient is fully passive;

**Phase two** The second cycle, patient is active and the robot is resistive;

**Phase three** Future exercises, both patient and robot are active.

The first step of this protocol consists of calculating the subject-related constant parameters. This concerns axis offset, inertial parameters, passive stiffness, and passive damping. The axis offset is defined as a constant and the inertial parameters are measured directly from a subject. Passive stiffness and damping were measured from the first exercise where the force and position were recorded from the totally passive patient. The second step consists of determining the remaining components of wrist model including the interaction torque. The subject must perform the desired movements providing the torque necessary to move the robot in the desired direction. In this case, the robot applied a resistive torque to calculate the torque produced by the patient. However, this step concerned only the patients who were in a middle or high level of rehabilitation progress. Indeed, patients who could not provide a minimum readable torque began with passive exercises and their wrist model contained only passive components. In addition, generated torque is a time variant parameter due to muscle fatigue. Therefore, fatigue estimation must be performed based on a reliable natural interface such as EMG signals. In future exercises, the total torque is the sum of the torque produced by the robot and the torque, if any, provided by the patient taking into consideration the fatigue constraints. The two main goals of wrist modeling are: (1) to evaluate the rehabilitation state level and (2) to extract the main components of the wrist dynamic model.

#### 2.3.4. IoT-Based Control Architecture

**Figure 12:**
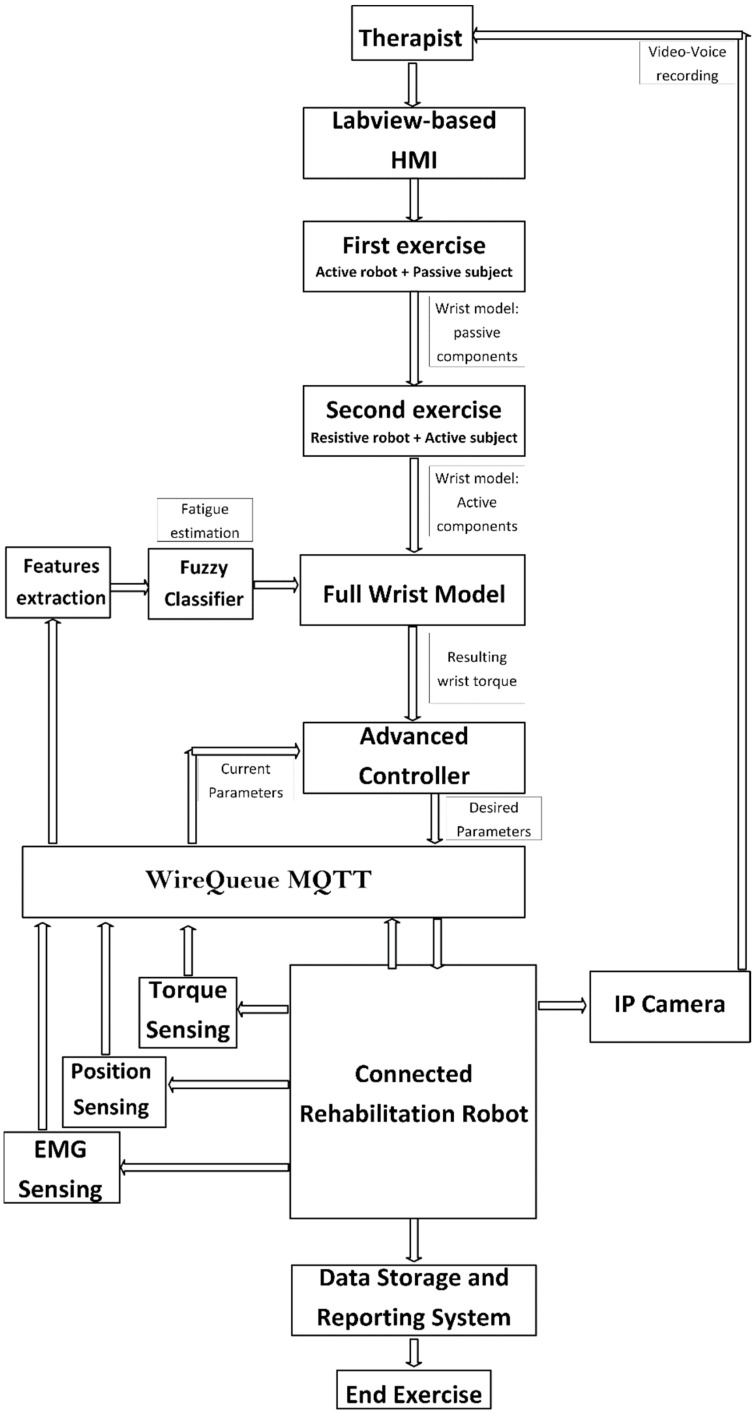
Operating system flowchart.

The physiotherapist starts the control procedure by entering the desired settings. The needed parameters are transmitted to the advanced controller block implemented in the LabVIEW-based HMI after the appropriate processing. The wrist robot receives the computed parameters, including the needed torque and desired position, through the MQTT protocol. The sb-RIO board processes the data from the current sensor, EMG sensor, and encoder before sending it back to the control station over the MQTT protocol. Then, the extracted frequential characteristics from the collected EMG rows are used to estimate muscle fatigue using extraction techniques. The EMG characteristics are sent into the fuzzy system, which estimates the patient’s fatigue. The control motion block uses position feedback to make the proper choice. The settings and the results are recorded in the database. Figure 12 depicts the operational flowchart of the system. 

**Figure 13:**
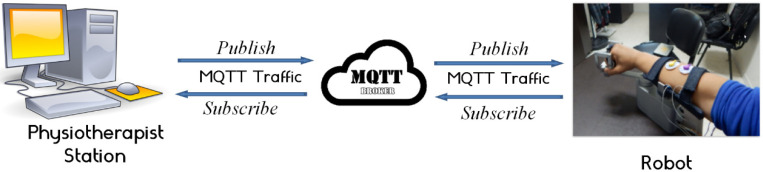
IoT platform.

The real-time component of the MQTT protocol is the most essential feature that truly differentiates it; a requirement that eliminates delay issues. To avoid disruptions caused by the increased demand for data interchange, network usage must be kept to a minimum. The MQTT protocol reduced the size of data packets, allowing the network to be used more efficiently and avoiding network disruptions caused by heavy data demand. A message dispatcher is the MQTT central server (also known as a broker). This server allows IoT devices to subscribe to or publish messages. Topics are subscribed to and published by the devices. As indicated in Figure 13, the broker’s job is to handle data flow between various devices. The function of the physiotherapist is to serve as a broker in the designed architecture. The calculated torque is the topic to publish in this circumstance. The wrist robot, on the other hand, is subscribed to this subject, allowing it to be alerted of any updates. 

## 3. Results and Discussion

**Figure 14:**
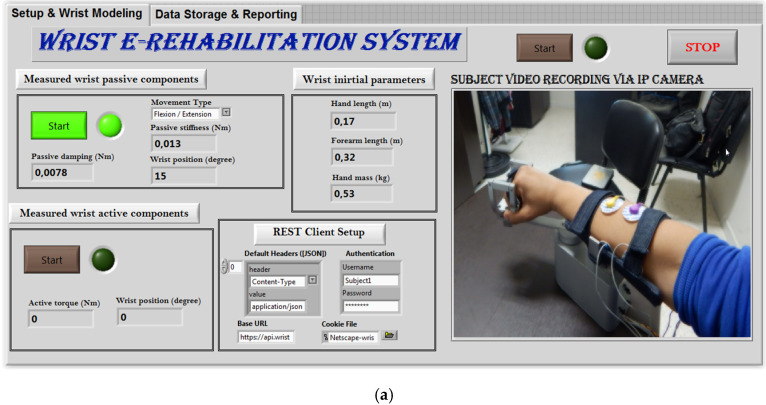

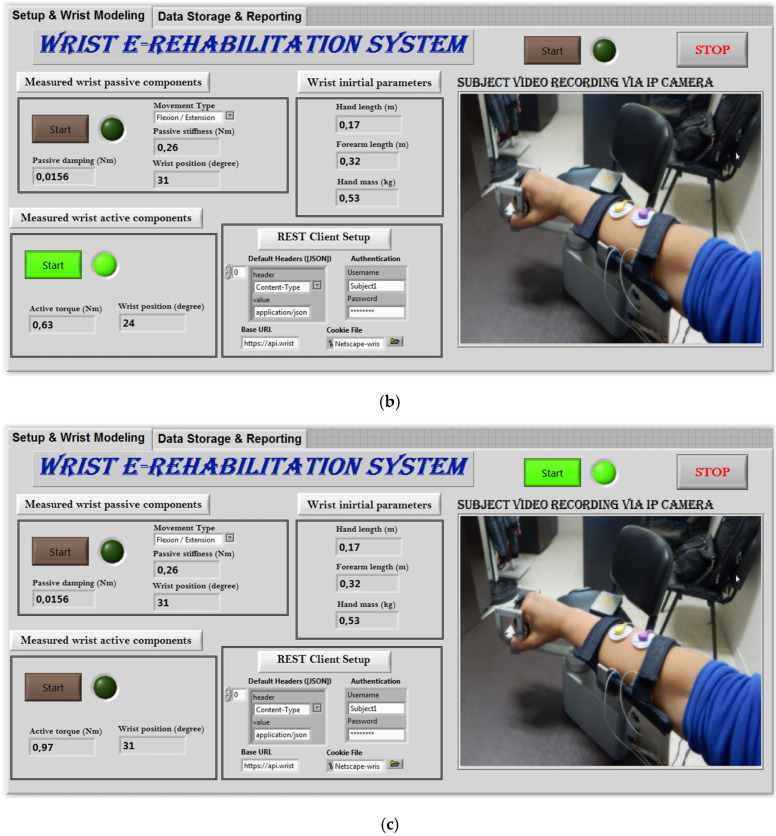
Setup sequences: (**a**) measuring passive wrist components; (**b**) measuring wrist active components; (**c**) continuous exercises.

The designed robot is light, practical, and user-friendly, making it an excellent choice for at-home rehabilitation. The fact that it is managed remotely by a physiotherapist makes it simpler for the patient, who has no involvement in the device’s operation. In reality, the patient is usually unable to accomplish anything after a stroke and is frequently inactive. This newly created device has a user-friendly HMI with two separate control displays. The first display serves as a configuration and supervision tool (Figure 14). The interface offers an authentication service at this stage. The security of the networked system is enhanced by this service. The system provides real-time streaming, as shown in Figure 14. The designed control architecture uses an embedded IP camera to feed videos to the router. A real-time video of the patient during the rehabilitation procedure is displayed on the physiotherapist’s PC connected to the router. 

Before beginning, the physiotherapist must input the wrist initial parameters of a related patient including hand length, forearm length and hand mass (Figure 14). Noting that these parameters are automatically stored in the related database and will be imported later in the next exercises. The physiotherapist can update these parameters anytime some things are changed. 

The next step consists of measuring the wrist passive components (Figure 14a) by launching the first exercise. The physiotherapist should select the exercise type as flexion/extension movement or ulnar/radial derivation. In this phase, the robot may perform some iterations to determine the passive components of the fully passive patient. The number of iterations is mainly related to the convergence of the computed data. The user will be informed visually by the convergence of this data. The final values of the passive components are stored in the connected database for assessment. 

After finalizing the passive component measurements, the next step consists of measuring the active components of the wrist joint (Figure 14b). In this phase, the active torque as well as the achieved range of motion are recorded. In this case, the rehabilitation robot applies a resistive torque and the patient is fully active. The patient is asked to perform the desired movement overcoming the resistive torque applied by the robot. The achieved range of motion and the maximum produced active torque are stored to evaluate the rehabilitation level. Passive and active components are both exploited in the control scheme during the continuous rehabilitation exercises (Figure 14c). 

**Figure 15:**
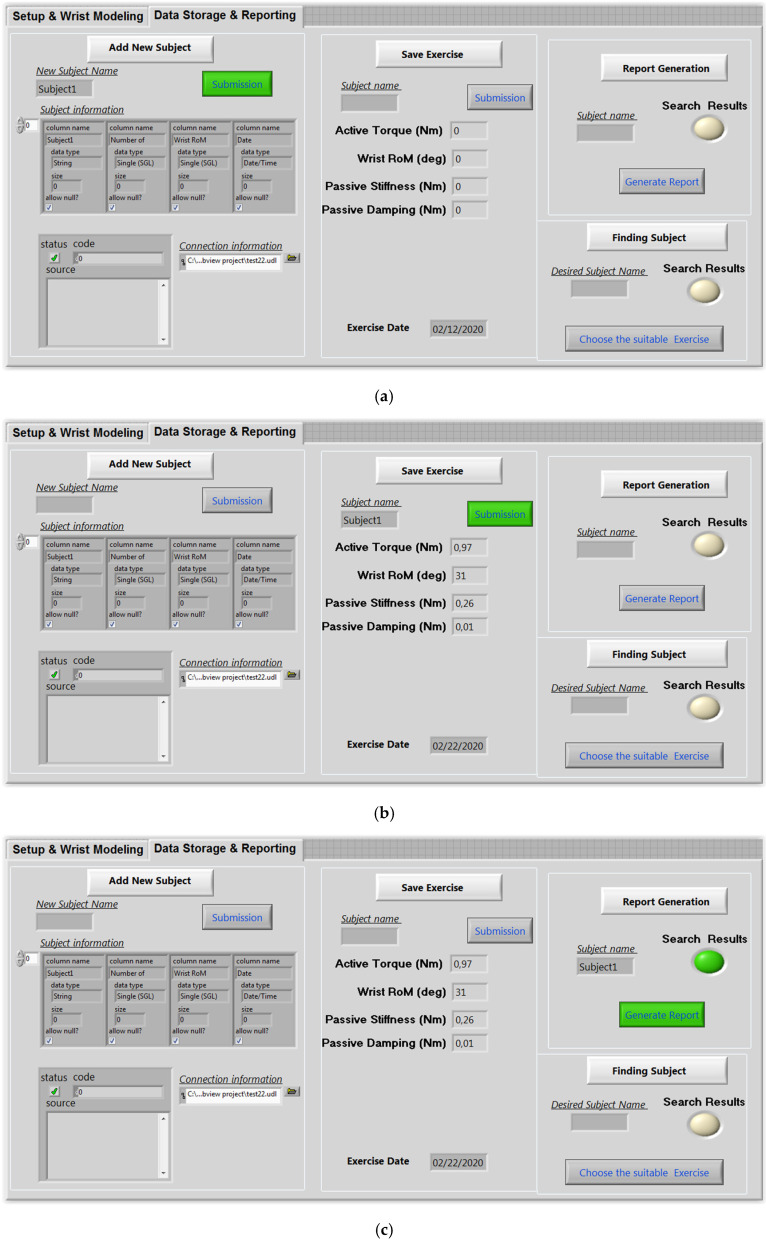
Data base interface: (**a**) Add new subject; (**b**) save exercise; (**c**) generate report.

**Figure 16:**
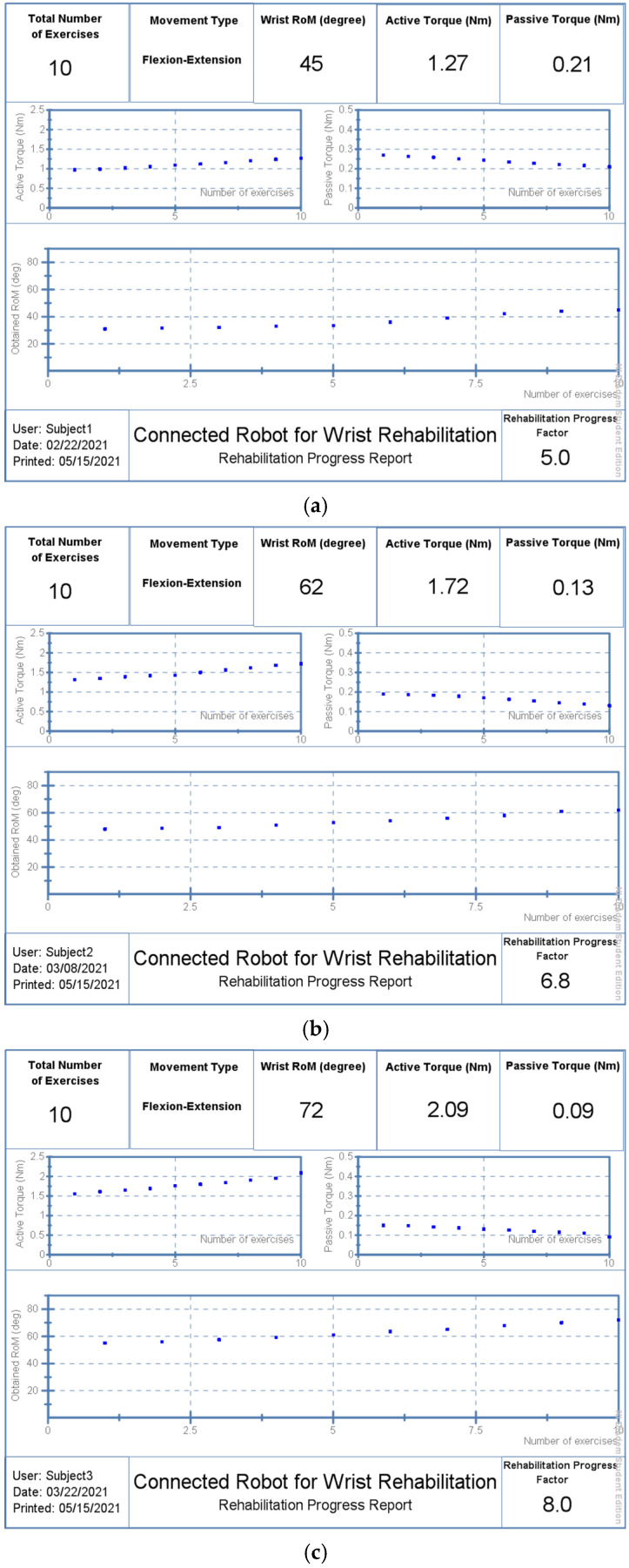
Generated reports: (**a**) Patient 1; (**b**) Patient 2; (**c**) Patient 3.

The second screen of the user interface presents the data storage and reporting interface. This screen provides some features that allow the physiotherapist to add a new subject (Figure 15a), to save a new exercise (Figure 15b), and to generate the related report (Figure 15c). Electronic health records can, in fact, provide several advantages for both doctors and patients. Patient records can be grouped and accessed remotely. As a result, it is simpler to move a patient from one rehabilitation clinic to another and to track the progress of their medical file. In this context, the software created, as shown in Figure 16, allows patients’ personal data, exercises, and patient follow-up reports to be recorded. 

Three participants, all with a wrist fracture, were tested just one week after their casts were removed. For ten days, the individuals undertook a comprehensive rehabilitation program to recover their abduction/adduction origin. A physiotherapist was in charge of the exercise, which started when the wrist model parameters were extracted. The exercise was performed three times for a total of 12 sets. The physiotherapist’s instructions were followed by the connected robot. The first tests on the three participants revealed that they had lost around 66 percent of their typical wrist RoM.

Subject 1 was received on 12 February 2021, as shown in Figure 16a. He underwent a 10-day work protocol punctuated by a day of rest (5 days of work, 1 day of rest, 5 days of work). He obtained a RoM of 45 degrees on the last day (22 February 2021) with RPF equal to 5:10. This rate is calculated according to the maximum RoM that can be reached by a healthy subject, as shown in Table 2. 

Rehabilitation performances also include reducing passive stiffness and increasing the produced active torque. Patient 1 reduced passive stiffness from 0.26 Nm to 0.21 Nm. on the other hand, and he showed good ability to produce more active torque starting from 0.93 Nm to 1.27 Nm, achieved on the last day. Likewise, Patient 2 was received on 27 February 2021 and he underwent the same protocol. He completed his last session with a RoM of 62 degrees and a RPF of 6.8:10, as shown in Figure 16b, reducing the passive stiffness to 0.13 Nm and producing a 1.72 Nm of active torque. Whereas Patient 3 began his rehabilitation protocol on 19 March 2020 and managed to reach a RoM of 72 degrees on 29 March 2020 with an RPF of 8:10, as shown in Figure 16c. His passive stiffness was minimized to 0.09 Nm while the maximum produced active torque was increased up to 2.09 Nm.

## 4. Conclusions

The development of rehabilitation robotics has made significant progress over the past few decades. By using technological engineering solutions, specialists hope to promote the recovery of functions. In this context, here, we proposed a remote-controlled wrist rehabilitation robot. A new protocol based on three steps was proposed to identify wrist health status and dynamic parameters of the human–robot interaction. Fatigue during the rehabilitation session has a direct impact on the torque produced by a patient. The proposed approach takes this constraint into account and integrates a fuzzy logic controller to estimate the degree of fatigue and update the estimated torque value accordingly. A human–machine interface was developed to control the robotic solution and to provide database space for storage and reporting. Tests were carried out on three different patients and showed encouraging results. The next steps involve moving to clinical trials by testing the designed platform on more subjects. In the future, the modeling of a human wrist joint will be optimized by integrating machine learning techniques for more reliability of the model parameters. 

## Figures and Tables

**Table 1:** Mechanical components.

Block	Article Number	Piece Name	Metal	Quantity
Block A	1	Box	ALU AU4G	1
	2	LCD box	Steel C45	1
	3	Box cover	Steel C45	1
	4	Higher back arc support	Steel C45	1
	5	Lower back arc support	Steel C45	1
Block B	6	Drive disk	Steel C45	1
	7	Sliding bar	Steel C45	1
Block C	8	Forward arc	Steel C45	1
	9	Handhold	Steel C45	1
	10	Handhold support in L	Steel C45	2
	11	Handhold support	Steel C45	2
Block D	12	Higher front arc support	Steel C45	1
	13	Intermediate front arc support	Steel C45	1
	14	Lower front arc support	Steel C45	1
Block E	15	Back arc	Steel C45	1

**Table 2:** Wrist RoM for a healthy subject.

Mvt Number	Flexion	Extension	Radial	Ulnar
*RoM*	80–90	70–90	15	30–45

## Data Availability

The data that support the findings of this study are available within the article.

## References

[1] GWorld Health Organization Stroke, Cerebrovascular Accident. http://www.emro.who.int/health-topics/strokecerebrovascularaccident/index.html.

[2] Samaee S., Kobravi H.R. (2019). Predicting the occurrence of wrist tremor based on electromyography using a hidden Markov model and entropy based learning algorithm. Biomed. Signal Process. Control.

[3] Moghaddam M.M., Moshaii A.A., Niestanak V.D. (2019). Fuzzy sliding mode control of a wearable rehabilitation robot for wrist and finger. Ind. Robot..

[4] Tran V.D., Dario P., Mazzoleni S., Posteraro F. (2018). Wrist robot-assisted rehabilitation treatment in subacute and chronic stroke patients: From distal-to-proximal motor recovery. IEEE Trans. Neural Syst. Rehabil. Eng..

[5] Islam R., Zaman A.U., Brahmi B., Bouteraa Y., Wang I., Rahman M. (2021). Design and Development of an Upper Limb Rehabilitative Robot with Dual Functionality. Micromachines.

[6] Abdallah I., Bouteraa Y., Rekik C. Web-based robot control for wrist telerehabilitation. Proceedings of the IEEE 4th International Conference on Control Engineering Information Technology (CEIT).

[7] Charles S.K., Hogan N. (2010). Dynamics of wrist rotations. J. Biomech..

[8] Peaden A.W., Charles S.K. (2014). Dynamics of wrist and forearm rotations. J. Biomech..

[9] Kooij H., Keemink Q., Stienen A. (2018). Admittance control for physical human-robot interaction. Int. J. Robot. Res..

[10] Ben Abdallah I., Bouteraa Y., Rekik C. (2016). Kinect-Based Sliding Mode Control for Lynxmotion Robotic Arm. Adv. Hum.-Comput. Interact..

[11] Bouteraa Y., Ben Abdallah I., Alnowaiser K., Ibrahim A. (2021). Smart solution for pain detection in remote rehabilitation. Alex. Eng. J..

[12] Zhang J., Meng Q., Yang X. (2019). Virtual rehabilitation training system based on surface emg feature extraction and analysis. J. Med. Syst..

[13] Foroutannia A., Akbarzadeh T.M.-R., Akbarzadeh A. (2022). A deep learning strategy for EMG-based joint position prediction in hip exoskeleton assistive robots. Biomed. Signal Process. Control.

[14] Bednarczyk M., Omran H., Bayle B. (2022). EMG-Based Variable Impedance Control With Passivity Guarantees for Collaborative Robotics. IEEE Robot. Autom. Lett..

[15] Bouteraa Y., Abdallah I.B., Elmogy A. (2020). Design and control of an exoskeleton robot with emg-driven electrical stimulation for upper limb rehabilitation. Ind. Robot..

[16] Aabdallah I.B., Bouteraa Y., Rekik C. (2016). Design of smart robot for wrist rehabilitation. Int. J. Smart Sens. Intell. Syst..

[17] Matsui M., Higashi T., Iso N., Hachisuka K., Yamamoto I., Hachisuka A. (2018). Wrist rehabilitation robot system and its effectiveness for patients. Sens. Mater..

[18] Telegenov K., Zeinullin M., Tursynbek I., Omarkulov N., Shintemirov A. Preliminary mechanical design of NU-Wrist: A 3-DOF selfaligning Wrist rehabilitation robot. Proceedings of the IEEE International Conference on Biomedical Robotics and Biomechatronics (BioRob).

[19] Kim J.P., Kim K., Hwang C.H., Phan H.L., Koo K.I. (2019). Wrist rehabilitation system using augmented reality for hemiplegic stroke patient rehabilitation: A feasibility study. Appl. Sci..

[20] Merzouk A., Choquet D., Gayda M., Ahmaidi S. (2005). Assessment of skeletal muscle fatigue in men with coronary artery disease using surface electromyography during isometric contraction of quadriceps muscles. Arch. Phys. Med. Rehabil..

[21] Bouteraa Y., Ben Abdallah I., ElMogy A., Ibrahim A., Tariq U., Ahmad T. (2020). A Fuzzy Logic Architecture for Rehabilitation Robotic Systems. Int. J. Comput. Commun. Control.

[22] De Leva P. (1996). Adjustments to Zatsiorsky-Seluyanov’s segment inertia parameters. J. Biomech..

[23] Krebs H.I., Volpe B.T., Williams D., Celestino J., Charles S.K., Lynch D., Hogan N. (2007). Robot-Aided Neurorehabilitation: A Robot for Wrist Rehabilitation. IEEE Trans. Neural Syst. Rehabil. Eng..

[24] Gielen C.C., Houk J.C. (1984). Nonlinear viscosity of human wrist. J. Neurophysiol..

[25] Perreault E.J., Kirsch R., Crago P.E. (2004). Multijoint dynamics and postural stability of the human arm. Exp. Brain Res..

[26] Oskoei M.A., Hu H. (2008). Support Vector Machine-Based Classification Scheme for Myoelectric Control Applied to Upper Limb. IEEE Trans. Biomed. Eng..

[27] Neu C.P., Crisco J., Wolfe S.W. (2001). In vivo kinematic behavior of the radio-capitate joint during wrist flexion–extension and radio-ulnar deviation. J. Biomech..

[28] Oskoei M.A., Hu H. GA-based feature subset selection for myoelectric classification. Proceedings of the IEEE International Conference on Robotics Biomimetics.

[29] Qingju Z., Zhizeng L. Wavelet de-noising of electromyography. Proceedings of the IEEE International Conference on Mechatronics Automation.

[30] Phinyomark A., Phukpattaranont P., Limsakul C. (2012). Feature reduction and selection for emg signal classification. Expert Syst. Appl..

